# Clot Composition Profiling in Large Vessel Occlusion Stroke Via Radiomics

**DOI:** 10.1002/ana.78160

**Published:** 2026-01-16

**Authors:** Andres Gudino, Elena Sagues, Carlos Dier, Diego Ojeda, Samantha Saenz‐Hinojosa, Sebastian Sanchez, Ariel Vargas, Linder Wendt, Maria Belen Torres, Emily Garces, Alex Hanson, Navami Shenoy, Connor Aamot, Susan A. Walsh, Anil K. Chauhan, Gowri Anil‐Peethambar, Santiago Ortega‐Gutierrez, Jay Kinariwala, Mohamed Elshikh, Amir Shaban, Enrique C. Leira, Osorio Lopes Abath Neto, Malik Ghannam, Edgar A. Samaniego

**Affiliations:** ^1^ Department of Neurology Carver College of Medicine, University of Iowa Iowa City IA USA; ^2^ Department of Neurology University of Connecticut Farmington CT USA; ^3^ Department of Neurology Yale University New Haven CT USA; ^4^ Institution for Clinical and Translational Science, University of Iowa Iowa City IA USA; ^5^ Iowa Institute for Biomedical Imaging, University of Iowa Iowa City IA USA; ^6^ Department of Internal Medicine‐Hematology, Oncology University of Iowa Iowa City IA USA; ^7^ Department of Neurosurgery Carver College of Medicine, University of Iowa Iowa City IA USA; ^8^ Department of Radiology Carver College of Medicine, University of Iowa Iowa City IA USA; ^9^ Department of Epidemiology Carver College of Medicine, University of Iowa Iowa City IA USA; ^10^ Department of Pathology Carver College of Medicine, University of Iowa Iowa City IA USA

## Abstract

**Objective:**

Clot composition may offer insights into the mechanism of ischemic stroke. Radiomics, a noninvasive imaging technique, enables tissue characterization through radiomic features (RFs). We aimed to evaluate clot composition using radiomics on non‐contrast computed tomography (NCCT).

**Methods:**

In the first phase, we conducted a prospective study comparing RFs with histopathology in thrombi retrieved via mechanical thrombectomy (MT). Thrombi were imaged using micro‐computed tomography (micro‐CT) and analyzed histologically. Matched micro‐CT and histological slices identified red blood cells (RBCs) and fibrin‐rich regions. RFs were extracted, and multivariate logistic regression identified features associated with each component. Spearman's correlation was used to assess associations between RFs and percentage composition. The same clots were localized on pre‐MT NCCT, and RFs were extracted. Micro‐CT and NCCT RFs were correlated to enable histology‐informed interpretation. Receiver operating characteristic analysis evaluated the ability of NCCT RFs to discriminate clot composition. In the second phase, radiomics was applied to a retrospective NCCT dataset from patients with ischemic stroke with varying etiologies.

**Results:**

Ten thrombi were analyzed using micro‐CT. Total energy (odds ratio [OR] = 1.35, 95% confidence interval [CI] = 1.20–1.54, *p* < 0.001) and large dependence high gray level emphasis (OR = 1.18, 95% CI = 1.07–1.32, *p* = 0.01) were associated with RBCs and correlated with >70% RBCs composition on NCCT (Rho = 0.752 and Rho = 0.815). Subsequently, 150 NCCT scans were analyzed, including 50 cardioembolic, 50 large artery atherosclerosis (LAA), and 50 cryptogenic strokes. Radiomic analysis indicated RBCs‐predominant composition in 72% of cardioembolic, 30% of LAA, and 50% of cryptogenic clots.

**Interpretation:**

Radiomics is a promising, noninvasive technique for characterizing clot composition. ANN NEUROL 2026;99:1179–1188

Approximately 30% of acute ischemic strokes (AIS) do not have an identifiable source. Clot composition can offer insights of AIS etiology, especially analyzing the amount of red blood cells (RBCs) and fibrin predominance among clots.^1^, ^2^, ^3^ For example, Brinjikji et al analyzed clots retrieved from 1,350 patients and concluded that clots from a large artery atherosclerosis (LAA) have a higher mean RBCs density than clots from a cardioembolic source.^4^ Likewise, determining the composition of clots can also help assess the response to thrombolytics.^5^, ^6^, ^7^ For instance, Choi et al histologically analyzed 52 clots and concluded that clots primarily composed of RBCs are more likely to respond to intravenous thrombolysis.^6^ Whereas these studies rely on histological examination of clots retrieved during mechanical thrombectomy (MT), this approach is limited. Not all patients undergo MT, and clot extraction is not always feasible. Moreover, histological analysis of clot composition is not part of routine clinical care.

An ideal approach to clot evaluation would be using data acquired through routine stroke imaging, because it would allow for an acute determination of clot composition before treatment and would not change standard stroke workflow. Radiomics is a computational quantitative imaging analysis tool that enables voxel‐by‐voxel quantification of imaging features from computed tomography (CT). Radiomics extracts a large number of quantitative data from medical images, which can provide surrogate information of biochemical processes. Among these extracted voxel characteristics are signal intensity, coarseness, and over 100 additional radiomic features (RFs).^8^, ^9^ We aimed to characterize clot composition by using micro‐CT to correlate extracted RFs with histological components of clots retrieved after MT, to further analyze clot composition using radiomics on images obtained during AIS work‐up.

## Methods

The study adhered to the STROBE checklist, was conducted under institutional review board (IRB) and consisted of 2 parts (Supplementary Fig S1). Patient consent was obtained for the first part of the analysis, whereas the IRB waived patient consent for the second part of the study. The first part involved a prospective cohort comparing RFs with pathological specimens in thrombus extracted with MT for the treatment of ischemic stroke. The RFs associated with specific clot components on micro‐CT were then matched to equivalent features identified on non‐contrast CT (NCCT) images. Once clot composition was inferred through radiomic analysis, we conducted the second part of the study, where NCCT scans were biologically interpreted through radiomics and correlated with stroke etiology from a large cohort of patients.

For the first part of the analysis, each of the retrieved clots were subjected to high‐resolution micro‐CT and histological analysis to quantify the core components, including RBCs and fibrin. Other cellular elements, such as leukocytes and platelets, were not analyzed, as they are more difficult to isolate using noninvasive imaging techniques like NCCT and partially contribute to the broader clot milieu dominated by RBCs and fibrin.^10^ Region‐based matching was then used to correlate the RFs that were extracted from micro‐CT with the histological composition of the clots. For quality control, two in vitro clots with known composition—one with RBCs (≥90%) and the other with fibrin (≥90%)—were scanned using the same micro‐CT protocol, and RFs were extracted from predefined regions of interest (ROIs) for comparison and validation.

The validated RFs associated with each histological component were then used to analyze NCCT images of the same 10 patients, to assess their reproducibility in clinical practice, with the aim to identify RBCs or fibrin rich clots, determined by a composition of >70% of either of these components. To date, there is no consensus definition for when a thrombus becomes “rich” in a specific component. We selected a 70% threshold because it approximates the midpoint of values reported in the literature, where clot composition thresholds for RBC‐rich or fibrin‐rich thrombi typically range from 60% to 90%.^10^, ^11^ The RFs most strongly correlated with RBCs or fibrin were then used to analyze NCCT scans from a broader cohort of patients with diverse stroke etiologies. The objective was to determine the predictive accuracy of these RFs in inferring clot composition from NCCT and, by extension, estimating the underlying stroke etiology based on imaging biomarkers.

### Radiomics Extraction

Segmentations across all imaging modalities were performed using 3D Slicer (version 5.6.1). RFs extraction was conducted using the PyRadiomics add‐in integrated into the 3D Slicer interface. For NCCT, RFs were extracted from images in their native Hounsfield unit scale, as these units represents a physically standardized measure of X‐ray attenuation that is inherently comparable across scans acquired under identical parameters. Because all NCCTs were obtained on the same scanner using a uniform acquisition protocol, additional intensity normalization was not applied. Micro‐CT grayscale values are not inherently standardized and can vary with detector gain, beam energy, and reconstruction settings. Therefore, voxel intensities were normalized using minimum–maximum scaling to the 0 to 1 range to account for scanner‐dependent differences and ensure comparability across specimens. Gray‐level discretization was applied using a fixed bin width of 25 for NCCT and 0.02 for micro‐CT, parameters selected to maintain texture stability and reproducible contrast resolution. Following extraction, all features were analyzed in their native scale to ensure consistent biological interpretation and reproducible thresholding across datasets.^8^, ^9^, ^12^, ^13^

### Histological Imaging Validation With In Vivo and In Vitro Clots

**FIGURE 1:**
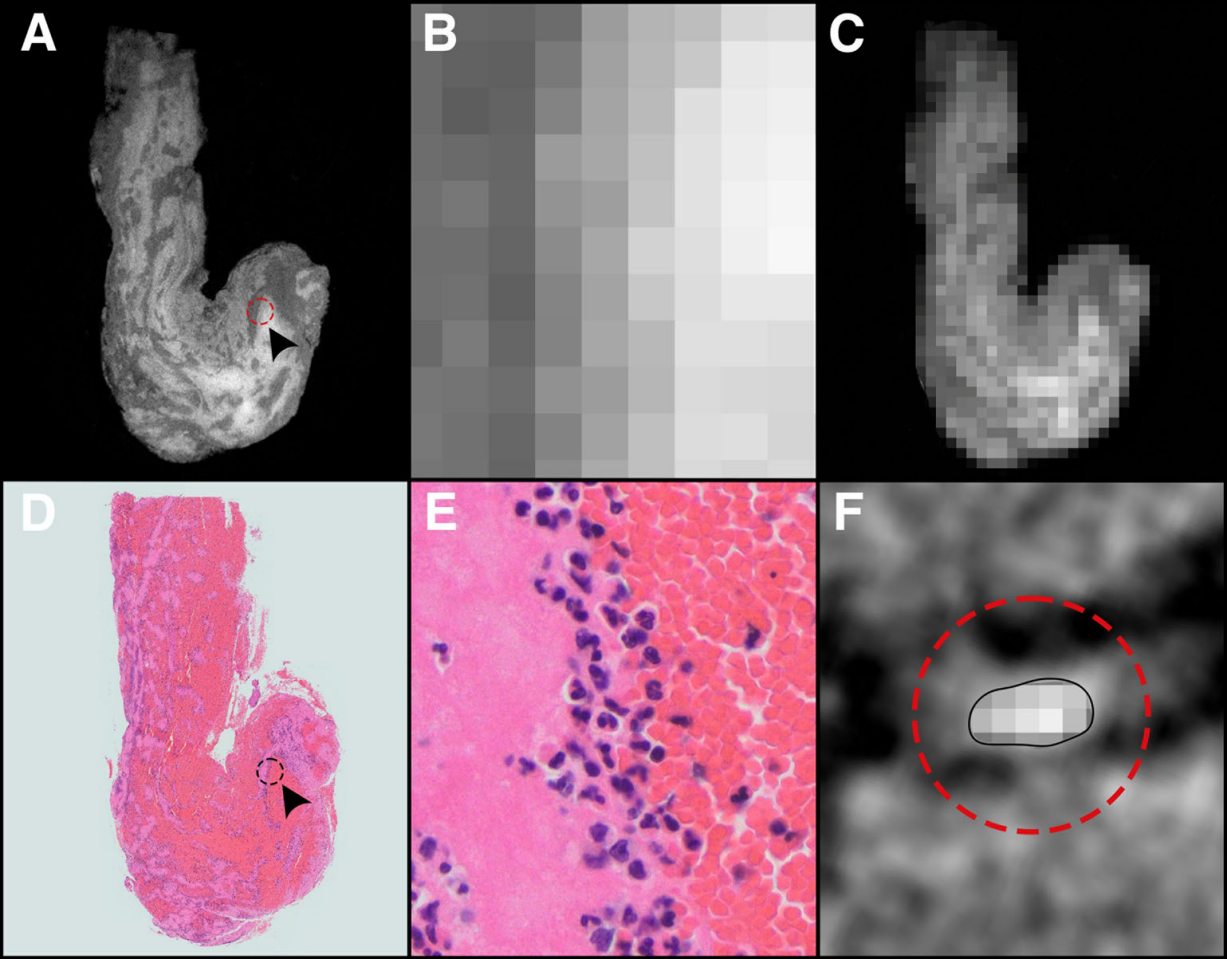
Micro‐CT and histological correlation. (A) Following mechanical thrombectomy, 10 clots were imaged on micro‐CT and sectioned for histological analysis. The micro‐CT slides were matched with corresponding histological homogeneous ROI (*arrowheads*). (B, C) RFs at a voxel‐level were extracted from micro‐CT images and correlated with their histological counterparts (D, E). E From left to right, fibrin (*pink*), white blood cells (*purple*), and RBCs (*dark red*), were identified. (F) The RFs obtained through this analysis were then correlated with RFs of NCCT ROIs of the 10 clots. Micro‐CT = micro‐computed tomography; NCCT = non‐contrast computed tomography; RBCs = red blood cells; RFs = radiomic features; ROIs = regions of interest. [Color figure can be viewed at www.annalsofneurology.org]

Ten clots retrieved after MT were imaged using high‐resolution micro‐CT and subsequently underwent histological analysis (Supplementary Material). Each clot was scanned using micro‐CT at 5 μm isotropic voxel resolution and subsequently sectioned at 5 μm thickness for histological analysis. Co‐registration was implemented to secure accurate alignment. Histological landmarks were used to match the histological section with the corresponding micro‐CT slice. Orthogonal‐plane alignment was iteratively refined until precise correspondence between histological images and micro‐CT was achieved (Supplementary Fig S2). RFs were extracted from ROIs of RBCs and fibrin based on established micro‐CT intensity thresholds, in which micro‐CT hyperintense regions are characterized by RBCs and hypointense regions are characterized by fibrin (Fig 1).^14^, ^15^ Once specific ROIs for RBCs and fibrin were identified in micro‐CT images, RFs were extracted. To validate these associations, two in vitro clots—one with ≥90% RBCs and one with ≥90% of fibrin—were scanned using the same micro‐CT protocol. RFs were extracted and compared to those from the in vivo specimens to confirm consistency in feature profiles (Supplementary Fig S3).

### Micro‐CT Association With NCCT

RFs were correlated with histological clot composition using different thresholds, 50 to 59% and 60 to 69% of RBCs and fibrin. A sensitivity analysis was additionally conducted on histological sections identified on micro‐CT with >70% composition of either RBCs or fibrin for radiomics analysis with NCCT (see Supplementary Fig S2). This approach ensured that only micro‐CT RFs strongly correlated with histologically validated RBCs or fibrin sections, were considered for correlation with NCCT‐derived RFs. The analysis was conducted on the NCCT scans of the same 10 clots used for histological evaluation. The NCCTs were acquired prior to MT at the time of large vessel occlusion (LVO) diagnosis and had not been exposed to iodine‐based contrast agents, thereby minimizing potential RFs contamination. For similar reasons, computed tomography angiographies (CTAs) were not analyzed. NCCTs were selected as the main imaging modality for analysis because it is part of standard of care in AIS. Cutoff values for each RF associated with >70% RBCs or fibrin composition were derived from this cohort.

### NCCT Imaging Analysis

Stroke etiology was independently determined by 2 blinded vascular neurologists (authors M.G. and E.A.S.) using the TOAST classification. It was categorized as cardioembolic, large artery atherosclerosis (LAA), or cryptogenic based on discharge summaries and follow‐up data.^16^ ROIs of clots were identified on NCCT using contrast cutoff from CTA to determine the LVO. This process was performed in axial, sagittal, and coronal planes, followed by morphological refinements and manual contour corrections to exclude adjacent vessel wall and bone. All segmentations were reviewed in 3 planes before feature extraction. Three raters (authors A.G., E.S., and C.D.) performed independent segmentations, and 2 vascular neurologists (authors M.G. and E.A.S.) adjudicated discrepancies to generate a consensus mask. The clots were subsequently isolated, and RFs were extracted (Supplementary Fig S4).

Only LVOs in the terminal internal carotid artery (ICA), the first segment of the middle cerebral artery (M1), or basilar artery (BA) were included. Cases that had prior contrast administration before NCCT were excluded due to potential contamination and artifact in the processing of RFs.^17^ The previously validated RF cutoff values obtained during the first part of the study, and associated with RBCs‐ and fibrin‐rich composition, were applied to this NCCT imaging cohort to estimate the predominant clot composition. NCCT and CTA acquisition parameters are detailed in Supplementary Table S1.

### Statistical Analysis

Statistical analyses were performed using R software version 4.3.3. For the first part of the analysis, micro‐CT RFs from RBCs and fibrin regions were summarized using medians and interquartile ranges. Differences among components were assessed using Wilcoxon rank sum tests. For each clot component, multivariate logistic regression models were built using forward stepwise selection based on Akaike Information Criterion, with a maximum of 4 predictors to avoid overfitting. An additional multivariate logistic regression model was conducted to identify RFs associated to RBCs and fibrin from our in vitro RBCs and fibrin rich clots. These models served as a feature‐selection step to identify RFs independently associated with RBCs and fibrin. The retained RFs were then correlated with histological percentage composition. Spearman correlations were used to link micro‐CT RFs with histological sections with >70% of RBCs and fibrin. RFs showing strong correlation (Rho > 0.600 and *p* < 0.05) were then compared to NCCT RFs from clots with >70% of the respective component. We selected Rho > 0.6 as the cutoff for strong correlation because this threshold has been used in radiomic studies to define meaningful feature associations.^18^, ^19^ An exploratory ROC analysis was performed to assess diagnostic performance, including area under the curve (AUC), sensitivity (SN), specificity (SP), positive predictive value (PPV), and negative predictive value (NPV); optimal cutoff values were determined using the Youden index. For the second part of the analysis, kappa statistics were used to determine agreement of stroke etiology between the 2 adjudicators. Intraclass correlation coefficient (ICC) using 2‐way random effects was conducted for clots segmentation in NCCT. Kruskal‐Wallis tests were used to assess RF differences across stroke etiologies in our broader NCCT dataset. The *p* values < 0.05 were considered statistically significant throughout the article due to the exploratory nature of the analysis.

## Results

### Histological Imaging Validation

Between November 2023 and January 2024, clots were prospectively collected from 10 consecutive patients with LVO who underwent MT (Supplementary Table S2). Clots were extracted in one piece, and without fragmentation. Demographic data are summarized in Table 1. Of the 10 clots retrieved after MT, 8 of 10 (80%) were in the M1 segment, 1 of 10 (10%) was in the ICA terminal, and 1 of 10 (10%) was in the BA. Three clots were predominantly composed of RBCs (clots 3, 4, and 9), and 2 were fibrin‐rich (clots 6 and 8). One clot contained calcium (clot 6). The remaining 5 clots had a mixed composition of RBCs and fibrin (Table 2).

**TABLE 1:** Patient Population

	Micro‐CT and Histological Correlation (N = 10)	NCCT Imaging Analysis (N = 150)
Clinical variables		
Age in yr, mean ± SD	61 ± 11	72 ± 14
Biological sex		
Men, N (%)	9 (90)	71 (47.3)
Women, N (%)	1 (10)	79 (52.6)
Race/ethnicity		
African American, N (%)	0 (0)	6 (4)
Hispanic, N (%)	0 (0)	8 (5.3)
White, N (%)	10 (100)	136 (90.6)
Medical history		
Hypertension, N (%)	3 (30)	129 (86)
Hyperlipidemia, N (%)	5 (50)	96 (64)
Diabetes, N (%)	2 (20)	36 (24)
Current smoker, N (%)	6 (60)	48 (32)
NIHSS at presentation, mean ± SD	14 ± 9	15 ± 6
Etiology		
Cardioembolic, N (%)	6 (60)	50 (33.3)
LAA, N (%)	2 (20)	50 (33.3)
Cryptogenic, N (%)	2 (20)	50 (33.3)
LVO		
ICA Terminal, N (%)	1 (10)	25 (16.6)
M1, N (%)	8 (80)	120 (80)
BA, N (%)	1 (10)	5 (3.3)

BA = basilar artery; ICA = internal carotid artery; M1 = first segment of the middle cerebral artery; LAA = large artery atherosclerosis; LVO = large vessel occlusion; micro‐CT = micro‐computed tomography; NCCT = non‐contrast computed tomography imaging; NIHSS = National Institute of Health Stroke Scale; SD = standard deviation.

**TABLE 2:** Histological Analysis of Clots

Clot	Composition			
	RBCs (%)	Fibrin (%)	WBCs (%)	Calcium (%)
1	52.19	41.39	6.41	–
2	56.06	34.57	9.36	–
3	83.08	14.38	2.53	–
4	71.18	23.01	5.80	–
5	41.58	53.63	4.78	–
6	6.46	80.48	3.02	10.03
7	65.00	30.73	4.26	–
8	11.90	81.80	6.29	–
9	88.52	9.85	1.62	–
10	50.30	43.9	5.79	–

RBCs = red blood cells; WBCs = white blood cells.

Radiomic analysis revealed that RBCs were associated with higher total energy (odds ratio [OR] = 1.35, 95% confidence interval [CI] = 1.20–1.54, *p* = < 0.001) and large dependence high gray level emphasis (LDHGLE; OR = 1.18, 95% CI = 1.07–1.32, *p* = 0.001). Fibrin was associated with higher 10th percentile values (OR = 2.01, 95% CI = 1.12–3.89, *p* = 0.025; Table 3).

**TABLE 3:** Radiomic Features for Each Clot Component

Component	Radiomic Features	OR	95% CI	*p*
RBCs	Total energy	1.35	1.20, 1.54	**< 0.001**
	LDHGLE	1.18	1.07, 1.32	**0.001**
	Joint average	0.93	0.90, 0.97	**< 0.001**
	Coarseness	0.83	0.61, 1.05	0.200
Fibrin	10th Percentile	2.01	1.12, 3.89	**0.025**
	Minimum	0.47	0.24, 0.86	**0.019**
	Long run low gray level emphasis	1.08	1.01, 1.18	0.076
	Run length non‐uniformity	2.42	0.79, 8.10	0.130

Note: The *P* values in bold represent statistical significance.

CI = confidence interval; LDHGLE = large dependence high gray level emphasis; OR = odds ratio; RBCs = red blood cells.

The same RFs associated with RBCs that were extracted from the 10 MT retrieved clots, were identified in the in vitro RBCs clot: total energy (OR = 1.64, 95% CI = 1.31–1.93, *p* = < 0.001) and LDHGLE (OR = 1.52, 95% CI = 1.23–1.74, *p* = < 0.001). Similarly, the same RFs identified for fibrin from the 10 MT retrieved clots were identified in the in vitro fibrin clot: 10th percentile (OR = 2.61, 95% CI = 1.54–4.8, *p* = < 0.001; Supplementary Table S3).

### Micro‐CT and NCCT Correlation

No significant correlations were observed between micro‐CT RFs and RBCs or fibrin composition within the 50 to 59% or 60 to 69% thresholds (Supplementary Table S4). On sensitivity analysis, micro‐CT total energy (Rho = 0.752, *p* < 0.001) and LDHGLE (Rho = 0.815, *p* < 0.001) were strongly correlated with histological sections that had more than 70% of RBCs. No correlation was found between micro‐CT 10th percentile and histological sections with more than 70% of fibrin (Supplementary Table S5). Given this lack of association, fibrin was excluded from further analysis in the study.

**FIGURE 2:**
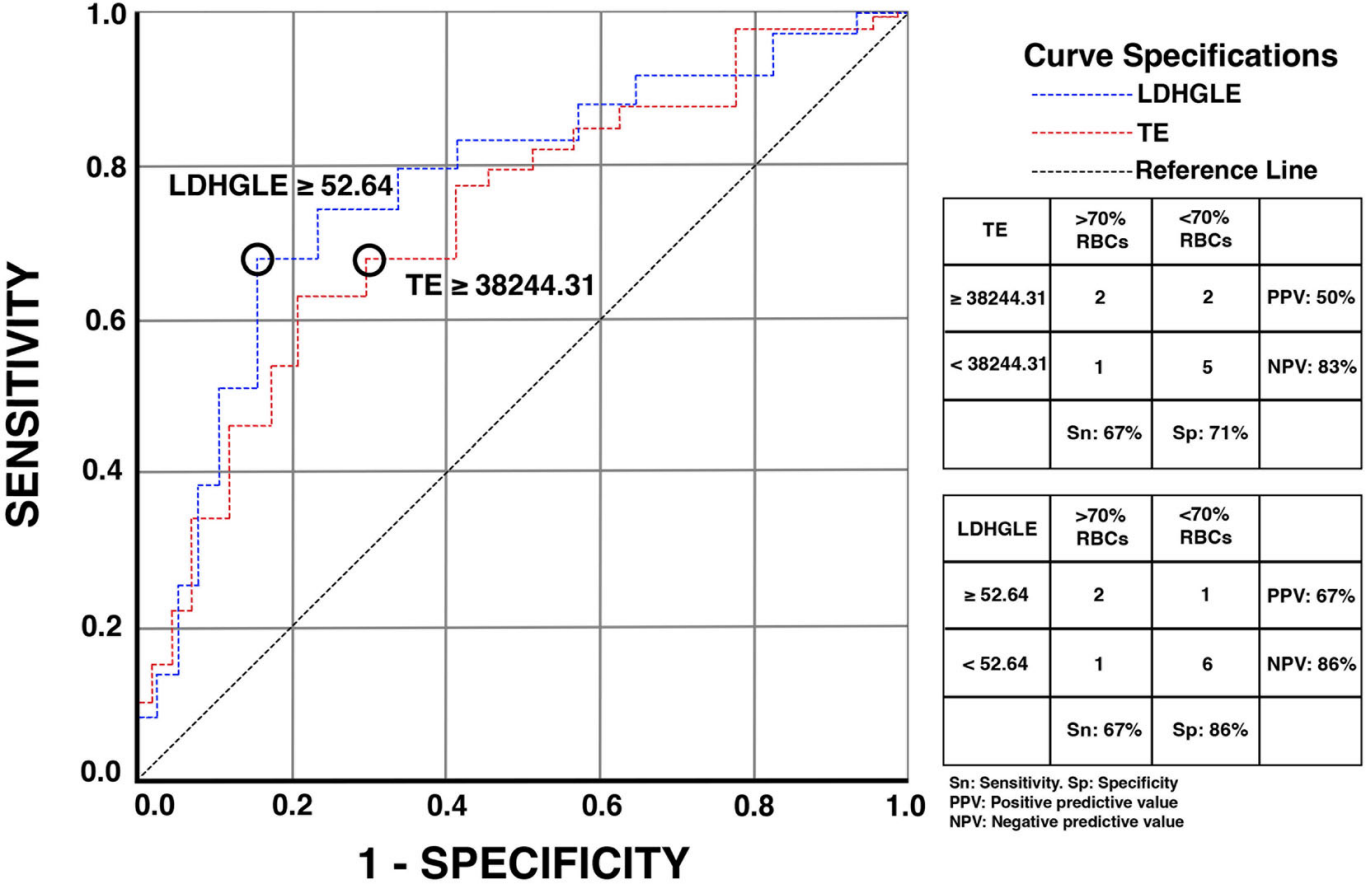
Exploratory receiver operating curve analysis of histology‐informed radiomic features. AUC, SN, SP, PPV, and NPV for detecting RBCs’ composition greater than 70% in NCCT using radiomic features TE and LDHGLE. Given the small sample size, this analysis is exploratory and intended only to provide preliminary thresholds for evaluating clot composition. AUC = area under the curve; LDHGLE = large dependence high gray level emphasis; NCCT = non‐contrast computed tomography; NPV = negative predictive value; PPV = positive predictive value; RBCs = red blood cells; SN = sensitivity; SP = specificity; TE = total energy. [Color figure can be viewed at www.annalsofneurology.org]

Micro‐CT total energy and micro‐CT LDHGLE were strongly correlated with NCCT total energy (Rho = 0.657, *p* = 0.002) and NCCT LDHGLE (Rho = 0.687, *p* = < 0.001) of clots that had more than 70% of RBCs per histological analysis (clots 3, 4, and 9). ROC analysis revealed that total energy (AUC = 0.700, SN = 67%, SP = 71%, PPV = 50%, NPV = 83%, and threshold = 38,244.31) and LDHGLE (AUC = 0.770, SN = 67%, SP = 86%, PPV = 67%, NPV = 86%, and threshold = 52.64; Fig 2) were accurate in determining clots with an RBCs composition higher than 70% in NCCT (clots 3, 4, and 9).

### NCCT Imaging Analysis

Initially, 430 patients were included for the NCCT imaging analysis. However, 280 of 430 (65.1%) patients were excluded for the following reasons: 134 of 280 (47.9%) patients did not have an identifiable clot in the NCCT images; in 128 of 280 (45.7%) patients, contrast was administered before the first NCCT became available; and 18 of 280 (6.4%) patients had spontaneous recanalization. Ultimately, NCCT images from a total of 150 patients were analyzed (see Table 1). The 2 adjudicators achieved excellent agreement (K = 0.82) in determining stroke etiology among this cohort. Similarly, the ICC for clot segmentations in NCCT was excellent (ICC = 0.89, 95% CI = 0.81–0.92).

Of these 150 patients, 50 of 150 (33.3%) had a stroke of cardioembolic origin, 50 of 150 (33.3%) had a stroke due to LAA, and 50 of 150 (33.3%) had cryptogenic strokes. In 120 of 150 (80%) patients, the occlusion was in M1, in 25 of 150 (16.6%) it was in the terminal ICA, and in 5 of 150 (3.3%) it was in the BA.

**FIGURE 3:**
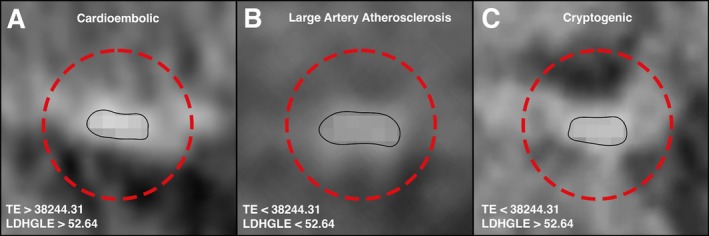
NCCT imaging analysis. Radiomics‐based analysis of clots from various stroke etiologies revealed distinct compositions. (A) Clots with TE and LDHGLE values exceeding the 70th percentile were predominantly rich in RBCs, a characteristic pattern observed in cardioembolic strokes. (B) In contrast, clots associated with large artery atherosclerosis exhibited TE and LDHGLE values below the established RBC‐rich threshold, indicating a lower RBC content. (C) Interestingly, cryptogenic clots demonstrated elevated LDHGLE values above the RBC threshold, suggesting a composition similar to cardioembolic clots, with a high proportion of RBCs. LDHGLE = large dependence high gray level emphasis; NCCT = non‐contrast computed tomography; RBCs = red blood cells; TE = total energy. [Color figure can be viewed at www.annalsofneurology.org]

The thresholds for determining clots with a predominant RBCs composition (>70%), total energy = 38,244.31, and LDHGLE = 52.64, were used in the characterization of the 150 clots chosen for analysis. Among cardioembolic, LAA, and cryptogenic clots, total energy (*p* < 0.001) and LDHGLE (*p* = 0.03) were significantly different among these etiologies. The total energy and LDHGLE thresholds identified RBCs‐rich clots (>70%) in 36 of 50 (72%) of cardioembolic, 15 of 50 (30%) of LAA, and 25 of 50 (50%) of cryptogenic clots (Fig 3 and Table 4).

**TABLE 4:** Radiomics and Stroke Etiologies

Radiomic Features	Cardioembolic (N = 50)	LAA (N = 50)	Cryptogenic (N = 50)	*p*
	N (%)	N (%)	N (%)	
Total energy	23 (46)	5 (10)	15 (30)	**< 0.001**
LDHGLE	30 (60)	12 (24)	21 (42)	**0.03**
Total energy + LDHGLE	36 (72)	15 (30)	25 (50)	**< 0.001**

Note: The *P* values in bold represent statistical significance (*p* < 0.05).

LAA = large artery atherosclerosis; LDHGLE = large dependence high gray level emphasis.

## Discussion

In this study, we aimed to characterize clot composition using a radiomics‐based approach. Because radiomics provides a voxel‐by‐voxel assessment, high‐resolution spatial analysis of clots can be performed while minimizing the confounding effects of thrombus composition heterogeneity. By precisely matching micro‐CT data with histological analysis, we identified RFs capable of accurately detecting RBCs in clots. We then applied these RFs to NCCT scans from the same patients, demonstrating high accuracy in identifying clots predominantly composed of RBCs. When radiomics analysis was conducted on a larger cohort of patients with stroke imaged with NCCT, we found that the majority of cardioembolic clots were RBC‐rich (>70% composition).

Micro‐CT offers ultra‐high spatial resolution (5 μm) and has previously been used to correlate imaging features with clot composition. Saghamanesh et al used micro‐CT to analyze 3 clots retrieved after MT, demonstrating that hyperintense regions of the clots corresponded with RBCs, whereas hypointense areas indicated fibrin, based on correlation with electron microscopy.^14^ Similarly, in our study, RBCs were predominantly located in regions with higher total energy and LDHGLE, which are associated with micro‐CT regions with higher signal intensity. Total energy is a first‐order RFs that quantifies the overall signal intensity, whereas LDHGLE reflects the spatial clustering of high‐intensity voxels. Thus, areas characterized by both higher signal intensity (total energy) and spatially coherent intensity patterns (LDHGLE) were strongly associated with RBCs‐rich regions. Therefore, RBC‐rich clots demonstrated higher total energy and LDHGLE reflecting dense erythrocyte packing and more uniform internal architecture generating higher voxel intensities and spatially coherent signal clusters on CT‐based imaging. Conversely, fibrin‐dominant thrombi have more heterogeneous, porous matrix with lower and more variable attenuation, which might explain why we did not find any correlation with radiomics.^8^, ^20^, ^21^ In contrast, Santo et al reported a strong correlation between total energy and white blood cells composition in their micro‐CT and histological analysis of 10 clots.^15^ However, our findings suggest that a higher total energy is associated with RBCs, not white blood cells. The discrepancy between studies may stem from differences in CT acquisition protocols and cohort characteristics. Notably, in Santo's study, it is unclear how many patients received iodinated contrast prior to NCCT, a known confounder in radiomics analysis. To avoid potential artifacts, we excluded all cases with prior iodine exposure. Similarly, Jiang et al reported that radiomics was more accurate in determining clot composition than known radiological features such as density, length, and enhancement sign.^22^

Clot composition analysis has been used to determine stroke etiology, predict the success of MT, and assess the efficacy of thrombolytics.^23^ Ideally clot composition should be assessed at the time of initial NCCT, prior to intervention, to inform stroke etiology and guide treatment strategy. Radiomics‐based profiling of NCCT images in our cohort revealed that 36 of 50 (72%) cardioembolic clots were predominantly composed of RBCs. However, prior studies have yielded inconsistent findings, likely due to methodological differences. For example, Ahn et al reported a higher fibrin content in cardioembolic clots, although over half of their cohort received thrombolysis, and nearly 90% of the clots were fragmented. Similarly, Liebeskind et al found no clear histological pattern associated with stroke etiology; their analysis relied on Hounsfield unit measurements to infer clot composition and was limited by clot fragmentation, as clot retrieval was performed using first‐generation MT devices.^24^, ^25^ In contrast, our study analyzed a large cohort of 150 NCCTs and studied intact clots prior to chemical or mechanical intervention, which may have contributed to a possible association between RBCs predominance and cardioembolic origin. During the validation phase of the study, clots extracted with MT were retrieved as non‐fragmented specimens. This aligns with findings by Minnerup et al who suggested that RBCs‐rich clots form under stasis conditions (common in atrial fibrillation), whereas fibrin‐rich clots arise from endothelial damage seen in LAA. Supporting this, Shin, Kim, and Sato et al have reported higher RBCs content in cardioembolic clots and greater fibrin in LAA‐related clots. Notably, in our cohort, 50% of cryptogenic clots were RBC‐predominant, which may indicate that a subset of cryptogenic strokes could have an underlying cardioembolic origin.^10^, ^26^, ^27^, ^28^, ^29^, ^30^, ^31^

This study has several limitations. First, this is a single center study with potential biases in the adjudication of stroke etiology; however, the adjudication was blinded and the 2 adjudicators had excellent agreement in determining the stroke etiology. Likewise, the first phase of our study included a limited number of clots (n = 10). However, each specimen yielded 5 matched histology–micro‐CT–NCCT correlations, for a total of 50 independent datapoints, providing a robust histopathologic basis for radiomic feature selection. Accordingly, the subsequent ROC analysis performed on the 10 clots in NCCT was considered exploratory and was primarily designed to identify preliminary threshold values for these histology‐informed RFs. Second, our 10 clots imaged using micro‐CT and analyzed histologically did not have a significant percentage composition of leukocytes and calcium; therefore, these components were not analyzed in detail on micro‐CT and NCCT. Similarly, other clot constituents, such as von Willebrand factor and platelets, were not analyzed, as their percentage contributions were too low to be reliably identified on NCCT. Nonetheless, as previous authors have mentioned, clots can be broadly divided into RBCs or fibrin clots.^10^ In a similar manner, our radiomics analysis did not identify a reproducible correlation with fibrin composition across multiple thresholds. This limitation is consistent with prior work showing that fibrin‐rich thrombi generate highly heterogeneous and low‐attenuation patterns on CT‐based imaging, making radiomic discrimination challenging.^20^ Third, a significant number of patients were excluded from the NCCT imaging analysis, as the ROIs used to determine the clot boundaries were highly selective. This was intended to minimize the sampling of non‐clot components in the NCCT images; thus, this step provided a detailed analysis of the ROIs with radiomics. Last, RFs can be influenced by CT acquisition parameters. To minimize this potential confounder, we ensured a close correlation between micro‐CT RFs and those derived from NCCT scans. Similarly, radiomic reproducibility across scanners and institutions remains a challenge, as variations in acquisition parameters may influence feature stability. The use of multi‐center datasets and standardized phantom‐based calibration protocols will be important next steps to harmonize radiomics and support broader clinical applicability. Finally, although NCCT is not a histologic gold standard, it was used to evaluate whether the histology‐informed RFs identified on micro‐CT can be detected on standard‐of‐care clinical imaging. Thus, the NCCT cohort served as a feasibility assessment rather than an independent biological validation set. However, external validation including different centers using standardized radiomics pipelines will be essential to support clinical adoption.

## Conclusions

In this proof‐of‐concept study, we found that radiomic analysis can identify RBC‐rich clots, potentially aiding in the determination of stroke etiology. In the future, radiomics could be incorporated into a fully automated pipeline capable of characterizing clots on NCCT and extracting histology‐informed radiomics in real time. This tool could be deployed as a plug‐in within advanced visualization software, facilitating rapid, noninvasive estimation of clot composition and potentially assisting in the determination of stroke etiology.

## Author Contributions

E.A.S, A.G contributed to the concept and design of the study. All authors contributed to the acquisition and analysis of data. E.A.S, E.C.L, A.G. contributed to drafting the text or preparing the figures. All authors approved the final version of the manuscript.

## Potential Conflicts of Interest

All the authors have no competing interests to disclose.

## Supporting information


**Supplementary Table S1.** Imaging parameters.
**Supplementary Table S2.** Patient data from the ten clots extracted for analysis.
**Supplementary Table S3.** Radiomic features in red blood cells and fibrin in‐vitro clots.
**Supplementary Table S4.** Correlation of micro‐computed tomography radiomic features with different composition thresholds.
**Supplementary Table S5.** Correlation of micro‐computed tomography radiomic features with > 70% of percentage composition.
**Supplementary Figure S1.** Study design. LAA = large artery atherosclerosis; LVO = large vessel occlusion; Micro‐CT = micro‐computed tomography; NCCT = non‐contrast computed tomography; RBCs = red blood cells; RFs = radiomics features; ROC = receiver operating characteristic.
**Supplementary Figure S2.** Correlative Analysis and Matching Methodology Between Histology and Micro‐computed Tomography (micro‐CT). This figure illustrates the process for precisely matching histological sections with corresponding micro‐CT slices to correlate radiomic features with clot composition. (A) A representative histological section is selected and accurately registered with (B) its corresponding micro‐CT slice. Matching is guided by shared topographic landmarks, such as tissue clefts and surface irregularities visible in both histology (*black arrows*) and micro‐CT (*white arrows*). Following the identification of distinct radiomic features (RFs) associated with specific components—namely red blood cells (RBCs) and fibrin, a whole‐slide micro‐CT segmentation is performed (C). These segmented RFs are subsequently correlated with the quantitative clot composition derived from the matched histology data. A sensitivity analysis is applied exclusively to histological sections that contain greater than 70% of a specific component to ensure robust correlation metrics. Panels D through I present representative examples of this correlation for the two primary clot types. (D) An RBC‐rich histological section is aligned with its matched micro‐CT slice (E) using the aforementioned topographical landmarks. The quantitative compositional correlation (F) confirms an 88.73% RBC content and 9.59% fibrin content. Conversely, (G) a fibrin‐predominant histological section is paired with its corresponding micro‐CT slice (H), yielding a high correlation (I) with 94.3% fibrin and 4.2% RBCs.
**Supplementary Figure S3.** Red Blood Cells and Fibrin Rich Clots. Micro‐computed tomography (micro‐CT) showing the signal intensity distribution of red blood cells (RBC, A) and fibrin (C) rich clots. Radiomics analysis depicted that RBCs regions (B) have higher signal intensity voxels (characterized by total energy) clustered between each other (depicted by large dependence high gray level emphasis). Fibrin regions (D) have lower signal intensity voxels (defined by 10th percentile).
**Supplementary Figure S4.** Clot localization and radiomics extraction. (A) (Left) Computed tomography angiography (CTA) and non‐contrast computed tomography (NCCT) images at admission were co‐registered. (Right) The clot was localized and segmented with the consequent radiomics features (RFs) extraction. Radiomics is a voxel‐by‐voxel tool that characterizes the signal intensity exhibited on imaging. The RFs were categorized by first‐order features, which quantify signal intensity on a histogram. The following textural features were also used: Gray‐level Co‐occurrence Matrix (GLCM), describes signal intensity interrelation throughout voxels; Gray‐level Run Length Matrix (GLRLM), considers consecutive voxels with similar signal intensity; Neighboring Gray Tone Difference Matrix (NGTDM), studies the voxel signal intensity relationship with surrounding voxels; and Gray‐level Size Zone Matrix (GLSZM), describes voxels with similar signal intensity, independent of their location.

## Data Availability

All data relevant to the study are included in the article or uploaded as Supporting Information.
