# Supplementary material for: Clot Composition Profiling in Large Vessel Occlusion Stroke Via Radiomics
Source: Ann Neurol. 2026 Jan 16;99(5):1179–88. doi: 10.1002/ana.78160 (PMC13092787; doi:10.1002/ana.78160)
Supplement: Supplementary file 1 — Supplementary Table S1. Imaging parameters. Supplementary Table S2. Patient data from the ten clots extracted for analysis. Supplementary Table S3. Radiomic features in red blood cells and fibrin in‐vitro clots. Supplementary Table S4. Correlation of micro‐computed tomography radiomic features with different composition thresholds. Supplementary Table S5. Correlation of micro‐computed tomography radiomic features with > 70% of percentage composition. Supplementary Figure S1. Study design. LAA = large artery atherosclerosis; LVO = large vessel occlusion; Micro‐CT = micro‐computed tomography; NCCT = non‐contrast computed tomography; RBCs = red blood cells; RFs = radiomics features; ROC = receiver operating characteristic. Supplementary Figure S2. Correlative Analysis and Matching Methodology Between Histology and Micro‐computed Tomography (micro‐CT). This figure illustrates the process for precisely matching histological sections with corresponding micro‐CT slices to correlate radiomic features with clot composition. (A) A representative histological section is selected and accurately registered with (B) its corresponding micro‐CT slice. Matching is guided by shared topographic landmarks, such as tissue clefts and surface irregularities visible in both histology (black arrows) and micro‐CT (white arrows). Following the identification of distinct radiomic features (RFs) associated with specific components—namely red blood cells (RBCs) and fibrin, a whole‐slide micro‐CT segmentation is performed (C). These segmented RFs are subsequently correlated with the quantitative clot composition derived from the matched histology data. A sensitivity analysis is applied exclusively to histological sections that contain greater than 70% of a specific component to ensure robust correlation metrics. Panels D through I present representative examples of this correlation for the two primary clot types. (D) An RBC‐rich histological section is aligned with its matched micro‐CT slice (E) [file ANA-99-1179-s001.docx]

**Supplementary material**

**Preparation of the 10 retrieved clots**

*Micro-computed tomography (micro-CT) imaging*

Ten clots that were retrieved immediately after MT were collected and stored in a petri dish in a solution of 10% phosphate-buffered formalin. The clots were then placed in a 50% Lugol’s iodine solution to enhance visualization of contrast differences, and after 24 hours they were taken for scanning. The micro-CT images were acquired on a ZEISS Xradia Versa 560. All scans were acquired at 80 kV, 6 W, with 1601 projections over 360°. The 0.4x objective was used to acquire the data, with an exposure time of 2.6 second per projection and a pixel size of approximately 5 μm. The data were reconstructed with Zeiss Scout and Scan software (version 16.1.14271) and calibrated to Hounsfield units.

*Histological evaluation*

After imaging, the clots were fixed in a series of 80%, 90%, and 100% ethanol solutions during 24-72 hours, followed by histological analysis. The clots were embedded in paraffin and 5-μm-thick sections and were transversely cut at fixed 200-μm intervals along the longest dimension of the clot to assure a complete analysis of the sample. Sections were collected on SuperFrost Plus (Fisher) slides, and hematoxylin and eosin (H&E) staining was performed. For whole slide imaging acquisition, mvSlide (Microvisioneer GmbH) was used, which generated scans at 40X magnification. The pixel classifier module from QuPath (version 0.5.0) was used for imaging analysis. The percentage of RBCs, fibrin, white blood cells, and calcium of each clot were calculated by measuring their respective pixel areas (in mm^2^), as determined by the trained pixel classifier, divided by the total pixel area of the clot. Between 7–10 H&E sections were analyzed per sample, and the corresponding percentage of components per slide was obtained. Finally, the average of all the slides was recorded to determine the entire clot composition.

**Supplementary Table 1. Imaging parameters**

|  | **NCCT** | **CTA*** |
| --- | --- | --- |
| **Scanner** | Siemens SOMATOM Force | |
| **Tube Voltage** | 120 kVp | 70 kVp |
| **Tube Current** | 320 mAs | 200 mAs |
| **Reconstructed voxel size** | 0.47 x 0.47 x 5.0 mm | 0.39 x 0.39 x 1.0 mm |
| **Slicing thickness** | 5.0 x 5.0 mm | 1.0 x 1.0 mm |

***** CTA was acquired after the administration of 40 ml ISOVUE-370 followed by 50 ml normal saline injected at 7 cc/s.

NCCT: Non-contrast computed tomography. CTA: Computed tomography angiography.

**Supplementary table 2. Patient data from the ten clots extracted for analysis.**

| ID | Age | Sex | Occlusion | Toast Etiology | HTN | HLD | DM2 | NIHSS at presentation | Time from onset to imaging | Key Findings |
| --- | --- | --- | --- | --- | --- | --- | --- | --- | --- | --- |
| 1 | 59 | M | M1 | Cardioembolic | Y | Y | N | 6 | 36 minutes | Atrial fibrillation on admission |
| 2 | 61 | M | M1 | Cardioembolic | N | N | N | 33 | 91 minutes | Atrial fibrillation on admission |
| 3 | 42 | M | M1 | Cryptogenic | N | N | N | 6 | 165 minutes | Stroke work-up was negative |
| 4 | 63 | M | M1 | Cryptogenic | N | N | Y | 8 | 75 minutes | Stroke work-up was negative |
| 5 | 66 | F | ICA Terminal | Cardioembolic | Y | Y | Y | 11 | 146 minutes | Presence of endocarditis on stroke work-up |
| 6 | 68 | M | M1 | Cardioembolic | N | N | N | 13 | 243 minutes | Atrial fibrillation on admission and history of heart failure with preserved ejection fraction |
| 7 | 72 | M | M1 | Cardioembolic | Y | Y | N | 15 | 199 minutes | Atrial fibrillation on admission and history of heart failure with preserved ejection fraction |
| 8 | 52 | M | M1 | Cardioembolic | N | Y | N | 6 | 68 minutes | Atrial fibrillation on admission and history of recent heart valve replacement |
| 9 | 55 | M | M1 | LAA | N | Y | N | 22 | 159 minutes | Radiographic evidence of atherosclerotic |
| 10 | 79 | M | Basilar | LAA | N | N | N | 21 | 20 minutes | Radiographic evidence of atherosclerosis |

ID: Identification. HTN: Hypertension. HLD: Hyperlipidemia. DM2: Diabetes Mellitus Type 2. NIHSS: National Institute of Health Stroke Scale. M1: First Segment of the Middle Cerebral Artery. ICA: Internal Carotid Artery. LAA: Large Artery Atherosclerosis.

**Supplementary Table 3. Radiomic features in red blood cells and fibrin in-vitro clots.**

| **Component** | **Radiomic features** | **OR** | **95% CI** | **p value** |
| --- | --- | --- | --- | --- |
| RBCs | Total Energy | 1.64 | 1.31, 1.93 | **<0.001** |
|  | LDHGLE | 1.52 | 1.23, 1.74 | **<0.001** |
|  | High Gray Level Emphasis | 1.39 | 0.79, 1.87 | 0.1 |
|  | Coarseness | 0.76 | 0.55, 1.3 | 0.18 |
| Fibrin | 10th Percentile | 2.61 | 1.54, 4.8 | **<0.001** |
|  | Minimum | 0.21 | 0.15, 0.43 | **<0.001** |
|  | Long Run Low Gray Level Emphasis | 1.05 | 0.98, 1.2 | 0.08 |
|  | Gray Level Non-Uniformity | 1.8 | 0.64, 1.73 | 0.3 |

RBCs: Red blood cells. LDHGLE: Large Dependence High Gray Level Emphasis. OR: Odds ratio. CI: Confidence interval.

**Supplementary Table 4. Correlation of micro-computed tomography radiomic features with different composition thresholds.**

| Radiomic Feature | 50-59% of red blood cells | | 60-69% of red blood cells | |
| --- | --- | --- | --- | --- |
|  | Rho | P-value | Rho | P-value |
| Total Energy | 0.325 | 0.285 | 0.412 | 0.180 |
| Large Dependence High Gray Level Emphasis | 0.380 | 0.224 | 0.450 | 0.157 |
| Joint Average | -0.125 | 0.674 | -0.212 | 0.489 |
| Coarseness | -0.183 | 0.519 | -0.259 | 0.412 |
|  | 50-59% of Fibrin | | 60-69% of Fibrin | |
|  | Rho | P-value | Rho | P-value |
| 10^th^ Percentile | 0.284 | 0.415 | 0.356 | 0.312 |
| Minimum | -0.231 | 0.546 | -0.259 | 0.478 |
| Long Run Low Gray Level Emphasis | 0.240 | 0.502 | 0.284 | 0.442 |
| Run Length Non-Uniformity | 0.183 | 0.643 | 0.321 | 0.401 |

**Supplementary Table 5. Correlation of micro-computed tomography radiomic features with >70% of percentage composition.**

| **Component** | **Radiomic features** | **Rho** | **p value** |
| --- | --- | --- | --- |
| Red Blood Cells | Total Energy | 0.752 | **< 0.001** |
|  | Large Dependence High Gray Level Emphasis | 0.815 | **< 0.001** |
|  | Joint Average | -0.244 | 0.433 |
|  | Coarseness | -0.431 | 0.120 |
| Fibrin | 10th Percentile | 0.581 | 0.093 |
|  | Minimum | -0.325 | 0.359 |
|  | Long Run Low Gray Level Emphasis | 0.312 | 0.298 |
|  | Run Length Non-Uniformity | 0.287 | 0.534 |


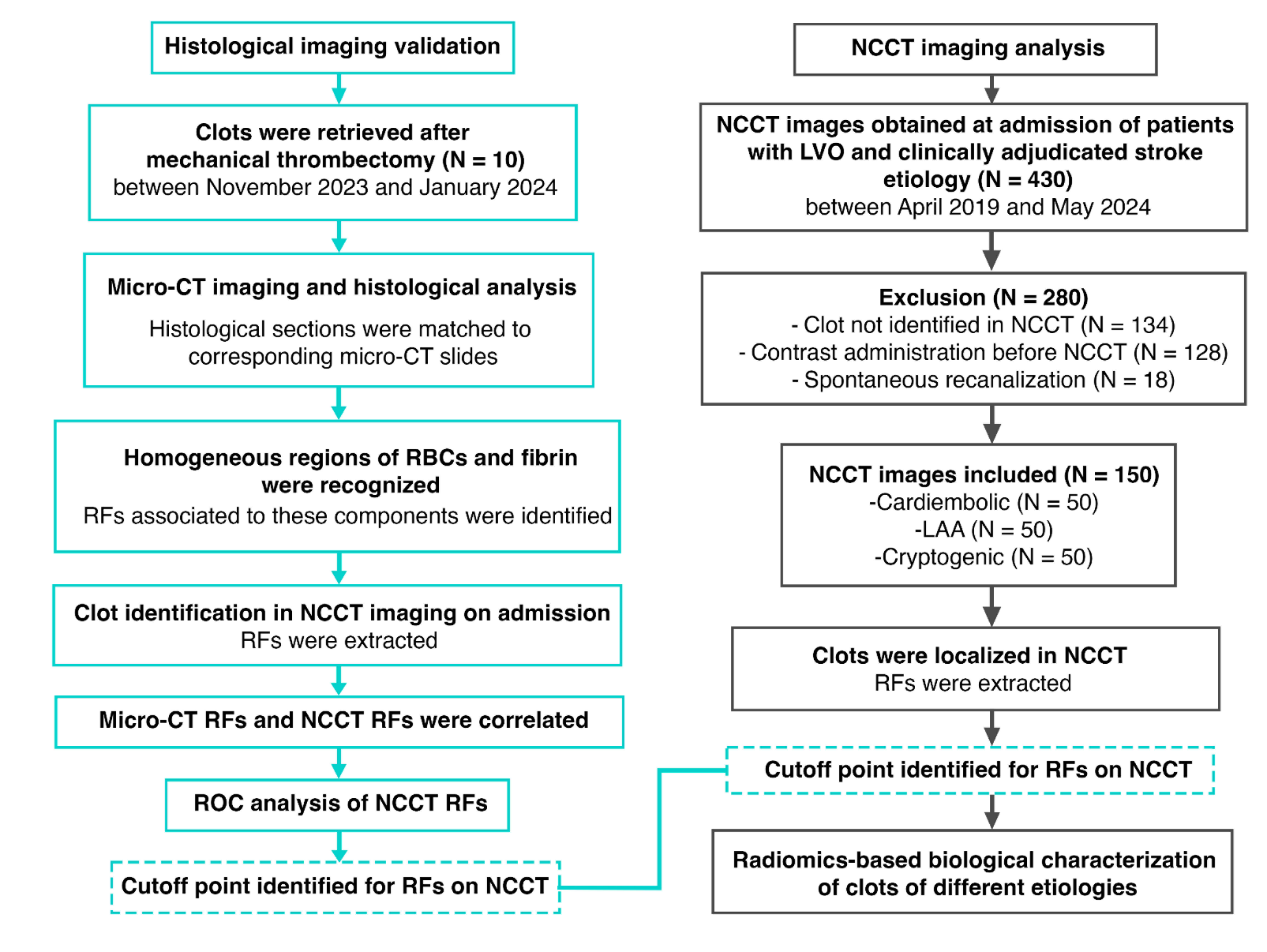


**Supplementary Figure 1. Study design.** Micro-CT: Micro-computed tomography. RBCs: Red blood cells. RFs: Radiomics features. NCCT: Non-contrast computed tomography. ROC: Receiver operating characteristic. LVO: Large Vessel Occlusion. LAA: Large artery atherosclerosis.


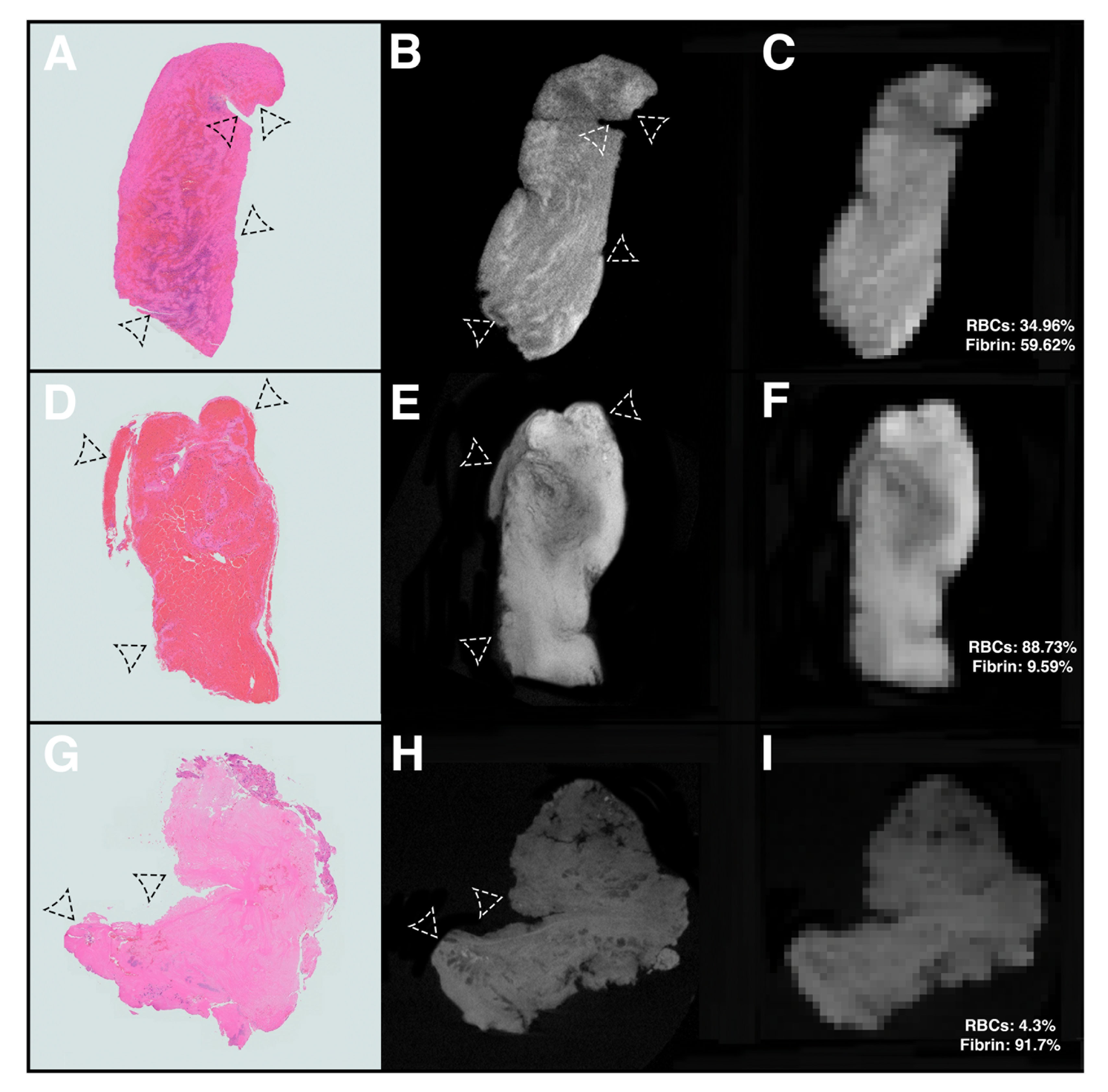


**Supplementary Figure 2. Correlation Between Histology and Micro-Computed Tomography (micro-CT).** This figure illustrates the process for precisely matching histological sections with corresponding micro-CT slices to correlate radiomic features with clot composition. (A) A representative histological section is selected and accurately registered with (B) its corresponding micro-CT slice. Matching is guided by shared topographic landmarks, such as tissue clefts and surface irregularities visible in both histology (black arrows) and micro-CT (white arrows). Following the identification of distinct radiomic features (RFs) associated with specific components—namely red blood cells (RBCs) and fibrin, a whole-slide micro-CT segmentation is performed (C). These segmented RFs are subsequently correlated with the quantitative clot composition derived from the matched histology data. A sensitivity analysis is applied exclusively to histological sections that contain greater than 70% of a specific component to ensure robust correlation metrics. Panels D through I present representative examples of this correlation for the two primary clot types. (D) An RBC-rich histological section is aligned with its matched micro-CT slice (E) using the aforementioned topographical landmarks. The quantitative compositional correlation (F) confirms an 88.73% RBC content and 9.59% fibrin content. Conversely, (G) a fibrin-predominant histological section is paired with its corresponding micro-CT slice (H), yielding a high correlation (I) with 94.3% fibrin and 4.2% RBCs.


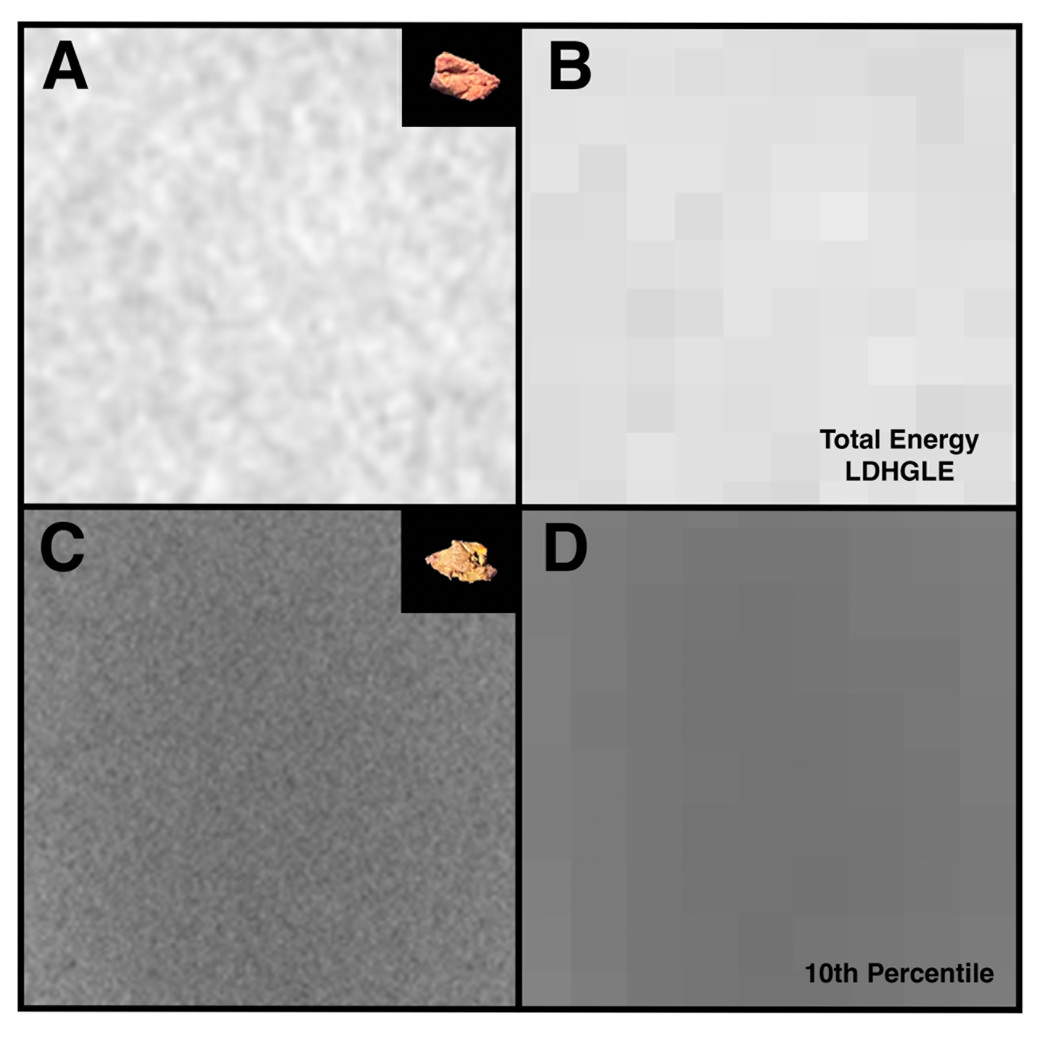


**Supplementary Figure 3. Red Blood Cells and Fibrin Rich Clots.** Micro-Computed Tomography (micro-CT) showing the signal intensity distribution of red blood cells (RBC, A) and fibrin (C) rich clots. Radiomics analysis depicted that RBCs regions (B) have higher signal intensity voxels (characterized by *Total Energy*) clustered between each other (depicted by *Large Dependence High Gray Level Emphasis*). Fibrin regions (D) have lower signal intensity voxels (defined by *10th Percentile* ).

**

**

**Supplementary Figure 4. Clot localization and radiomics extraction. (A)** (Left) Computed tomography angiography (CTA) and non-contrast computed tomography (NCCT) images at admission were co-registered. (Right) The clot was localized and segmented with the consequent radiomics features (RFs) extraction. Radiomics is a voxel-by-voxel tool that characterizes the signal intensity exhibited on imaging. The RFs were categorized by first-order features, which quantify signal intensity on a histogram. The following textural features were also used: Gray-level Co-occurrence Matrix (GLCM), describes signal intensity interrelation throughout voxels; Gray-level Run Length Matrix (GLRLM), considers consecutive voxels with similar signal intensity; Neighbouring Gray Tone Difference Matrix (NGTDM), studies the voxel signal intensity relationship with surrounding voxels; and Gray-level Size Zone Matrix (GLSZM), describes voxels with similar signal intensity, independent of their location.
